# Alzheimer’s Disease (AD): Depression is associated with lower Mini‐Mental State Exam (MMSE) Scores

**DOI:** 10.1002/alz.085244

**Published:** 2025-01-03

**Authors:** Caroline Komatsu

**Affiliations:** ^1^ Ministry of Health, São Paulo, 04207000 Brazil

## Abstract

**Background:**

Neuropsychiatric symptoms (NPS) are core features of AD and depression is a common co‐morbidity in people with AD, affecting overall outcomes and decreasing quality of life.

**Method:**

205 outpatients with the diagnosis of AD conforming criteria proposed by NINCDS‐ADRDA (2011), followed‐up at a public university hospital, had their Geriatrics Depression Scale – 15 (GDS ‐15) and MMSE scores evaluated.

**Results:**

125 patients (60,97%) were diagnosed with depression and 80 patients (39,03%) were not diagnosed with depression. From the depression group, 20 patients (16%) presented severe cognitive impairment (MMSE < 10), 50 patients (40%) presented moderate cognitive impairment (MMSE = 10 – 20) and 55 patients (44%) presented mild cognitive impairment (MMSE > 20). From the non‐depression group, 10 patients (12,5%) presented severe cognitive impairment, 30 patients (37,5%) presented moderate cognitive impairment and 40 patients (50%) presented mild cognitive impairment. Both groups, depression and non‐depression, had on average the same educational level: 3,02 and 3,42 years, respectively.

**Conclusion:**

In this sample of patients with AD, depression is associated with lower MMSE scores when compared to non‐depression group.

